# Chromosome-Level Analysis of the *Pelochelys cantorii* Genome Provides Insights to Its Immunity, Growth and Longevity

**DOI:** 10.3390/biology12070939

**Published:** 2023-06-30

**Authors:** Xiaoli Liu, Haiyang Liu, Yakun Wang, Mingzhi Li, Liqin Ji, Kaikuo Wang, Chengqing Wei, Wei Li, Chen Chen, Lingyun Yu, Xinping Zhu, Xiaoyou Hong

**Affiliations:** 1 Key Laboratory of Tropical and Subtropical Fishery Resources Application and Cultivation, Ministry of Agriculture and Rural Affairs, Pearl River Fisheries Research Institute, Chinese Academy of Fishery Sciences, Guangzhou 510380, China; liu_xiaoli1988@126.com (X.L.);; 2 Guangzhou Bio & Data Technology Co., Ltd., Guangzhou 510555, China; 3 College of Life Science and Fisheries, Shanghai Ocean University, Shanghai 201306, China

**Keywords:** *Pelochelys cantorii*, chromosome, genome, resources, longevity

## Abstract

**Simple Summary:**

The Asian giant soft-shelled turtle (*Pelochelys cantorii*), belonging to the order Testudines (family Trionychidae, genus *Pelochelys*), is one of the largest inland aquatic turtle species. However, due to excessive economic development, *Pc*. *cantorii* is critically endangered and rarely seen in the wild. As early as 1989, China listed the turtle as a key aquatic wildlife protection animal at the national level, but the conservation biology of *Pc*. *cantorii* has not been fully elucidated due to a lack of reference genomes. Here, based on a high-quality chromosome-level genome for *Pc*. *cantorii*, acquired by a combination of Illumina short-read, PacBio long-read and Hi-C scaffolding technologies in a previous study, we analyzed the evolutionary state of *Pc*. *cantorii*. Moreover, we found that several candidate genes associated with tumor suppression, growth and age were expanded, implicating their potential roles in the exceptional longevity of turtles. These findings will be an enabling resource for genetic and genomic studies to support fundamental insights into *Pc*. *cantorii* conservation.

**Abstract:**

The Asian giant soft-shelled turtle, *Pelochelys cantorii* (Trionychidae), is one of the largest aquatic turtles in China and was designated as a First-Grade Protected Animal in China in 1989. Previous investigation based on a combination of Illumina short-read, PacBio long-read and Hi-C scaffolding technologies acquired a high-quality chromosome-level genome of *Pc. cantorii*. In this study, comparative genomic analysis between *Pc. cantorii* and 16 other vertebrate genomes indicated that turtles separated from the ancestor of archosaurians approximately 256.6 (95% highest posterior density interval, 263.6–251.9) million years ago (Mya) (Upper Permian to Triassic) and that *Pc. cantorii* separated from the ancestor of *Pd. sinensis* and *R. swinhoei* approximately 59.3 (95% highest posterior density interval, 64.3–54.3) Mya. Moreover, several candidate genes, such as *VWA5A*, *ABCG2*, *A2M* and *IGSF1,* associated with tumor suppression, growth and age were expanded, implicating their potential roles in the exceptional longevity of turtles. This new chromosome-level assembly has important scientific value in the study of conservation of *Pc. cantorii* and also enriches the evolutionary investigation of turtle species.

## 1. Introduction

The Asian giant soft-shelled turtle (*Pelochelys cantorii*), belonging to the order Testudines (family Trionychidae, genus *Pelochelys*), is one of the largest inland aquatic turtle species. Decades ago, *Pc. cantorii* was widely distributed in Asia, in regions such as southeastern China, India, Bangladesh, Myanmar, Laos, Thailand, Cambodia, Vietnam, Malaysia, Indonesia and the Philippines [1]. In China, according to our investigation, the Asian giant softshell turtle was historically distributed in rivers, such as the Mekong River basin, the Pearl River, the Hanjiang River, the Minjiang River and the Oujiang River systems, which all drain into the ocean [2]. It has a long history and is of great scientific value in paleogeography and paleontological evolution. This species is also an important indicator of ecological health of the Pearl River in China [3]. However, due to excessive economic development and human consumption, *Pc. cantorii* is critically endangered and rarely seen in the wild. Though adult *Pc. cantorii* has a large body size and can grow up to 100 kg, the young individuals are easily captured in place of another well-known and closely related species, *Pelodiscus sinensis*, due to the similar appearance. Through extensive field resource surveys, so far, only 13 adult individuals are kept in captivity [2]. In order to prevent further reduction in its total population, as early as 1989, China listed the turtle as a key aquatic wildlife protection animal at the national level. The World Conservation Union approved *Pc. cantorii* as an endangered species in 2000, and it was later added to Appendix II of the Convention on International Trade in Endangered Species of Wild Fauna and Flora treaty in 2003 [3].

To strengthen the conservation and management of *Pc. cantorii*, government departments and scientific research institutions have increased the conservation biology research on *Pc. cantorii*. The Pearl River Fisheries Research Institute, Chinese Fishery Academy of Sciences successfully bred 10 *Pc. cantorii* hatchlings from sexually mature turtles (1 female and 1 male) in 2014. Thanks to the improvement of breeding technology of *Pc. cantorii*, from 2015 to 2021, more than 1000 juveniles that are currently 1–9 years old were cultured in the Gaoming breeding and protection base (Gaoming, Foshan, China) using four sexually mature turtles (two females and two males). Moreover, microsatellite multiplexes for *Pc. cantorii* were developed to evaluate the genetic diversity and structure of artificially assisted breeding generations [3]. The breakthrough of breeding technology of *Pc. cantorii* also received attention from the government administration. The Ministry of Agriculture and Rural Affairs of China established the “*Pelochelys cantorii* Rescue Action Plan (2019–2035)” in 2019. The Ministry of Agriculture and Rural Affairs of China then organized and carried out the first wild adaptation protection test for *Pc. cantorii* in 2020. A total of 20 juvenile turtles that are 4–5 years old and 1.04–1.66 kg were released in a reservoir in Gaoming, Foshan. To further track these individuals, each turtle was implanted with PIT chips. Subsequently, in 2022, two juvenile turtles captured in Taili Reservoir were identified as released individuals with a weight gain of 264.27% and 172.64%, respectively. The evaluation of the growth data and the physical state of the captured turtles revealed that the released juveniles had adapted to the wild environment after 20 months.

To enhance individuals’ knowledge about the reproduction of *Pc. cantorii*, Hong et al. reported the nesting behavior, clutch size, egg size, incubation period, as well as other reproductive characteristics of 4 adults (2♀, 2♂) under captive conditions from 2015 to 2017 [4]. Moreover, to fill in the blank in the study of the Asian giant soft-shelled turtle skeleton system, they described the morphological characteristics of the complete skeletal system of Asian giant soft-shelled turtles and included diagrams [5]. The skeletal structure of the Asian giant soft-shelled turtle and Chinese soft-shelled turtle had significant differences in snout length and the structure of third cervical spine. In terms of the entire cervical spine, the ratio of the spine length to the back armor length is 0.66 for the Asian giant soft-shelled turtle and 1.07 for the Chinese soft-shell turtle, which provide the basis for the identification of Asian giant soft-shelled turtles.

Third-generation genome sequencing technologies based on single-molecule reading have promoted the understanding and recognition of biological life activities [6]. However, because of the lack of the third-generation genome information, it is difficult for us to have a comprehensive understanding of the growth environment, ecological characteristics, biological characteristics and resource conservation of *Pc. cantorii*. Here, based on the high-quality chromosome-level genome assembly for *Pc. cantorii* (Scientific data under review), we analyzed the phylogenetic relationship of *Pc. cantorii* with other species. We also identified several candidate genes that are associated with tumor suppression, growth and longevity. The new chromosome-level assembly genome will be an enabling resource for genetic and genomic studies to support fundamental insights into *Pc. cantorii* biology.

## 2. Materials and Methods

### 2.1. Evolutionary and Comparative Genomic Analyses

In our previous study, to advance conservation research, we developed a chromosome-level genome assembly for *Pc. cantorii* (genome assembly data of *Pc. cantorii*, SRR24179425) based on a combination of Illumina short-read, PacBio long-read and Hi-C scaffolding technologies. To describe the genome evolution of *Pc. cantorii*, Orthofinder V2.4 software [7] was used to identify orthologous gene families by comparing the protein data of *Pc. cantorii* and 16 other genomes from previously reported vertebrates, including *Homo sapiens* (GCA_009914755.4), *Pelodiscus sinensis* (GCA_000230535.1), *Rafetus swinhoei* (GCA_019425775.1), *Trachemys scripta* (GCA_013100865.1), *Gopherus agassizii* (GCA_002896415.1), *Mauremys reevesii* (GCA_016161935.1), *Mauremys mutica* (SRR24179425), *Chrysemys picta* (GCA_000241765.5), *Platysternon megacephalum* (GCA_003942145.1), *Chelonia mydas* (GCA_015237465.2), *Xenopus. laevis* (GCA_017654675.1), *Gallus gallus* (GCA_016699485.1), *Danio rerio* (GCA_000002035.4), *Anolis carolinensis* (GCA_000090745.2), *Ornithorhynchus anatinus* (GCA_004115215.4) and *Alligator sinensis* (GCA_000455745.1). In all the phylogenetic analyses mentioned above, *D. rerio* was used as the outgroup species. The obtained gene families were annotated using the PANTHER V15 database [8].

MAFFT V7.205 [9] was used to compare the sequences of each single-copy gene family. Then, Gblocks V0.91b [10] (parameter: −5= H) was used to remove regions with poor sequence alignment or high differences, and finally, the amino acid sequences of each species were concatenated into a supersequence. Subsequently, the detection tool ModelFinder [11] was used to detect the model, and the best model was obtained as JTT + F + I + G4. Then, using the optimal model, the maximum likelihood (ML) method was used to construct the evolutionary tree using IQ-TREE v1.6.11 [12], where the bootstrap times were set to 1000.

### 2.2. Estimation of Divergence Times

Divergence time was estimated by the MCMCTree package of the PAML v4.9 program [13] under the relaxed clock model. Using the TimeTree (http://www.timetree.org/, accessed on 15 July 2021) website, the fossil time of *Ce. mydas* vs. *D. rerio* (440.0–423.4 Mya), *M. reevesii* vs. *M. mutica* (27.9–15.6 Mya), *Ce. mydas* vs. *T. scripta* (29.6–28.1 Mya), *X. laevis* vs. *M. reevesii* (355.7–348.4 Mya), *H. sapiens* vs. *Ga. gallus* (322.4–316.0 Mya), *An. carolinensis* vs. *Ga. gallus* (286.8–274.9 Mya), *Ga. gallus* vs. *M. reevesii* (266.2–252.6 Mya), *Pd. sinensis* vs. *Cr. picta* (181.6–146.9 Mya), *Cr. picta* vs. *Ce. mydas* (121.0–77.0 Mya), *M. reevesii* vs. *Cr. picta* (94.8–65.5 Mya), *R. swinhoei* vs. *Pc. cantorii* (66.7–54.5 Mya) and *Pd. sinensis* vs. *R. swinhoei* (56.9–49.2 Mya) were used to modify the fossil time obtained by the software based on the algorithm. Subsequently, the MCMCTree package of the PAML v4.9 program was used to estimate the parameters required by the divergence time, including gradient and Hessian. The correlated molecular clock and JC69 model were used to estimate divergence time based on the amino acid sequences of each species. Two repeated calculations were made to observe the consistency (the correlation between the two replicates in this experiment was 1). The iteration parameters of the Markov chain were as follows: sample number = 10,000,000; burn-in = 5,000,000; and sample frequency = 30. The final evolutionary tree with divergence time was visualized using MCMCTreeR v1.1 [14].

### 2.3. Collinearity Analysis

Patterns of collinearity can provide insight into the evolutionary history of a genome, and inform on potentially useful downstream analyses [15]. Moreover, collinearity analysis of genes can also be used as a means to verify the accuracy of genome assembly under the premise of a reference genome or related species genome [16]. Here, similar gene pairs were identified using Diamond V0.9.29.130 [17] to compare the gene sequences of *Ce. mydas*, *T*. *scripta*, *M*. *reevesii*, *Cr*. *picta*, *M*. *mutica*, *R*. *swinhoei* and *Ga*. *gallus*. Then, MCScanX [18] was used to determine whether similar gene pairs were near each other on chromosomes according to gff3 file, and finally, all the genes in colinear blocks were obtained. A colinear picture of the linear patterns of each species was drawn by JCVI V0.9.13 (https://doi.org/10.5281/zenodo.31631, accessed on 21 July 2021).

### 2.4. Duplicate Gene Analysis

Gene duplication is considered the primary source of new genes in Bacteria, Archaea and Eukaryota [19]. Numerous new functions have originated from gene duplication [20].

Here, the eggNOG 5.0 [21] database was used to annotate genes of over 17 vertebrates, then the numbers of “preferred_name” genes provided in the annotation results were counted. The genes with more copies in turtles were preferentially screened. To further explore the longevity mechanism of *Pc. cantorii*, several tumor-suppressor candidates were annotated based on the Tumor Suppressor Gene Database (https://bioinfo.uth.edu/TSGene/, accessed on 5 March 2022), and longevity-related genes were annotated based on the Ageing Gene Database (https://genomics.senescence.info/genes/index.html, accessed on 15 March 2022). Moreover, genes related to body size reported previously were used to annotate the selected candidate genes to obtain a clear definition of genes related to body size in *Pc. cantorii*.

### 2.5. Relative Synonymous Codon Usage (RSCU) Analysis

Here, the RSCU values of *Pc. cantorii* and 16 other genomes were calculated and obtained for each codon as previously described by Sharp and Li (1987) [22]. Heatmaps of the RSCU values were generated using the R package heatmap (each row was normalized by the R scale function).

## 3. Results

### 3.1. Genome Evolution

**Figure 1:**
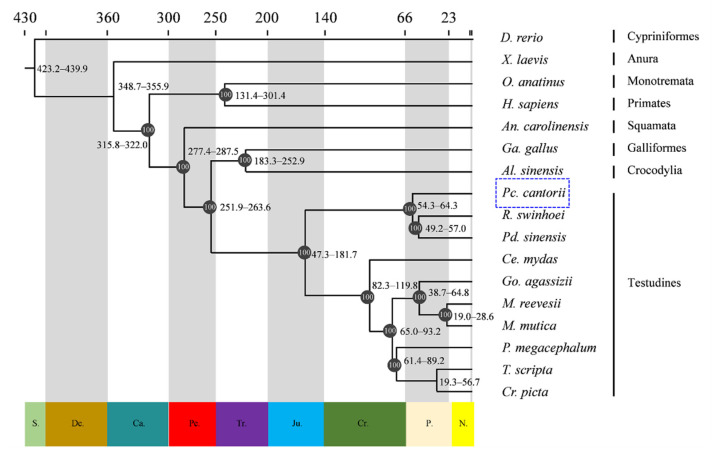
Phylogenomic relationships among 17 vertebrates. The numbers in the black circle represent bootstrap support values. The numbers on the branch represent the estimated divergence time with 95% confidence intervals. The number on the bottom of the tree is the geological time and the number at the top of the tree is the absolute age, in millions of years, as defined by the shadow of each geological period. C.: Cambrian; Pe.: Permian; Tr.: Triassic; Ju.: Jurassic; Cr.: Cretaceous; Pa.: Paleogene; N.: Neogene. The species in the blue dot square represents the Asian giant soft-shelled turtle.

In our previous study, a chromosome-level genome for *Pc. cantorii* (Genome assembly data of *Pc. cantorii*, SRR24179425) was developed. The assembled genome consists of 33 pseudochromosomes with a genome size of 2.16 Gb and a scaffold N50 length of 120.17 Mb. To further investigate the phylogenetic position of *Pc. cantorii*, the genomes of 17 vertebrate species, including 10 turtles (*T. scripta*, *Ce. mydas*, *Cr. picta*, *Pd. sinensis*, *R. swinhoei*, *M. mutica*, *M. reevesii*, *P. megacephalum*, *Go. agassizii* and *Pc. cantorii*) and seven other vertebrates (*X. laevis*, *Al. sinensis*, *Ga. gallus*, *O. anatinus*, *An. carolinensis*, *D. rerio and H. sapiens*), were compared. In total, the amino acid sequences of 1071 single-copy genes were identified among these 17 species. The ML phylogenetic tree shows that *Pc. cantorii* was most closely related to *Pd. sinensis* and *R. swinhoei*, together with those of other turtle groups, including *T. scripta*, *Ce. mydas*, *Cr. picta*, *M. mutica*, *M. reevesii*, *P. megacephalum* and *Go. agassizii*, which were sister crocodilians and birds (Figure 1). Analysis of time-constrained molecular clocks based on the fossil record shows that turtles separated from the ancestor of archosaurians approximately 256.6 (95% highest posterior density interval, 263.6–251.9) Mya. Hard- (*Pc. cantorii*, *Pd. sinensis* and *R. swinhoei*) and soft-shelled turtles (*T. scripta*, *Ce. mydas*, *Cr. picta*, *M. mutica*, *M. reevesii*, *P. megacephalum* and *Go. agassizii*) split 164.4 (95% highest posterior density interval, 181.7–147.3) Mya, while *Pc. cantorii* separated from *Pd. sinensis* and *R. swinhoei* approximately 59.3 (95% highest posterior density interval, 64.3–54.3) Mya (Figure 1).

### 3.2. Collinearity Analysis

**Figure 2:**
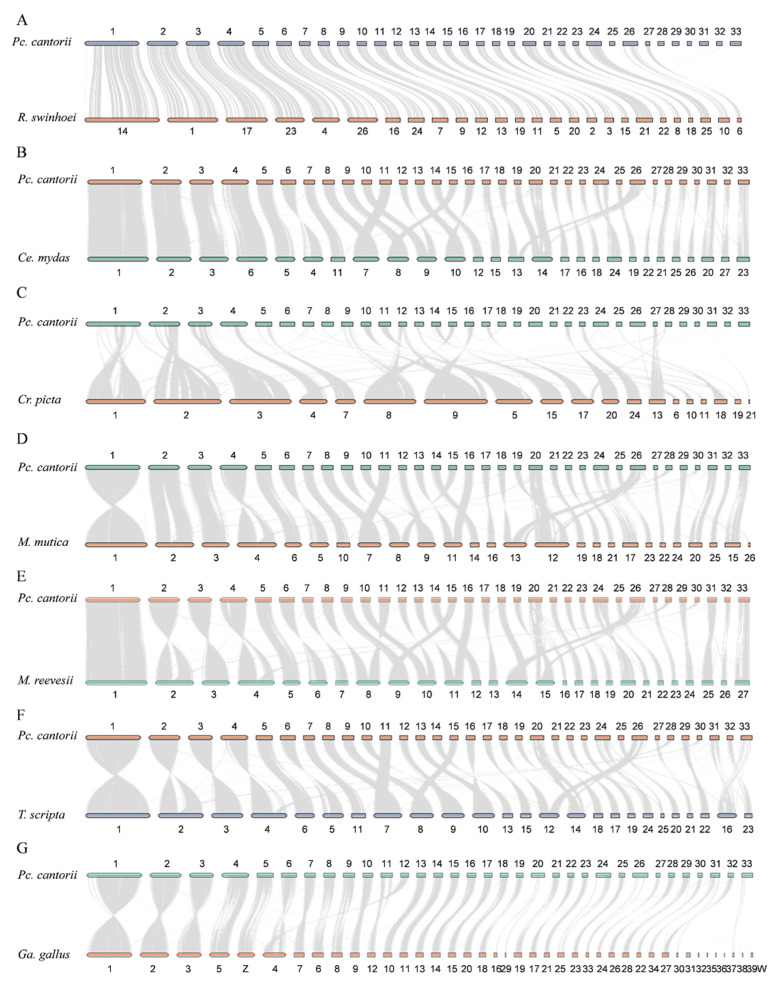
Chromosome-level synteny among *Pc. cantorii* and other species. (**A**): *Pc. cantorii* and R. swinhoei; (**B**): *Pc. cantorii* and *Ce. Mydas*; (**C**): *Pc. cantorii* and *Cr. picta*; (**D**): *Pc. cantorii* and *M*. *mutica*; (**E**): *Pc. cantorii* and *M. reevesii*; (**F**): *Pc. cantorii* and *T. scripta*; (**G**): *Pc. cantorii* and *Ga. gallus*.

Synteny of the *Pc. cantorii* assembly was compared to *R*. *swinhoei*, *Ce. mydas*, *Cr*. *picta*, *M*. *mutica*, *M*. *reevesii*, *T*. *scripta* and *Ga*. *gallus* (Figure 2). Although these seven turtle species have similar genome sizes, there is variation in their chromosome numbers: *R*. *swinhoei* (*n* = 26), *Ce. mydas* (*n* = 28), *Cr*. *picta* (*n* = 25), *M. mutica* (*n* = 26), *M*. *reevesii* (*n* = 27), *T. scripta* (*n* = 25) and *Pc. cantorii* (*n* = 33). Each chromosome in the *Pc. cantorii* genome can find homologous fragments on the chromosomes of other species except *R*. *swinhoei* (Figure 2A) and *Cr*. *picta* (Figure 2C). From the linear collinear graph (Figure 2), chromosome 1 is the largest chromosome in all these species except *Cr*. *picta*. For *Pc. cantorii* and *R*. *swinhoei*, twenty-six chromosomes showed a one-to-one relationship (Figure 2A), as well as a number of rearrangements of pairwise chromosomes between *Pc. cantorii* and the remaining six species (Figure 2B–G).

### 3.3. Relative Synonymous Codon Usage (RSCU) Analysis

**Figure 3:**
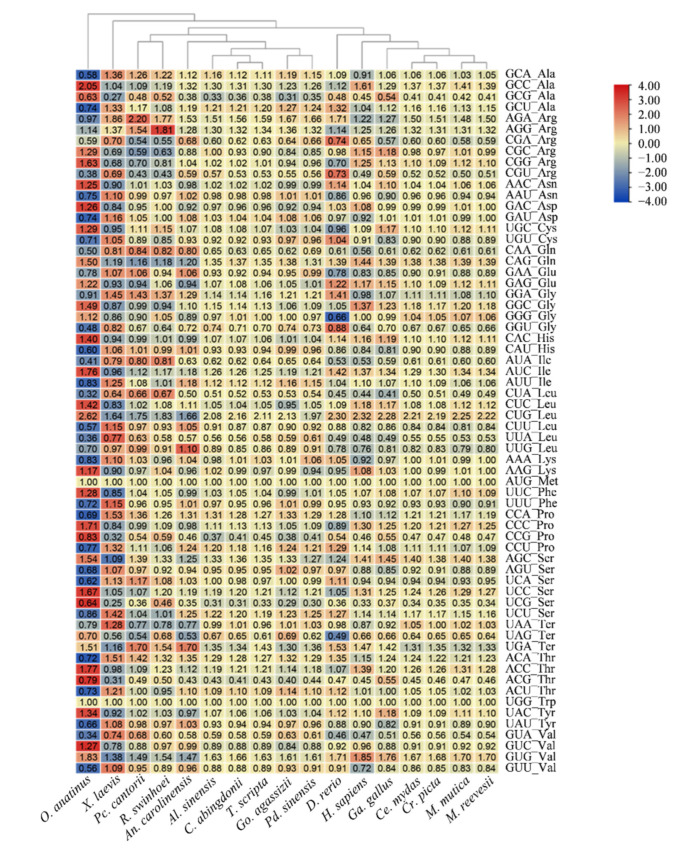
Relative synonymous codon usage (RSCU) among 17 phylogenomically analyzed vertebrates. Blue to red indicates the RSCU value from low to high.

Codon usage bias ubiquitously exists in animals and has been revealed to contribute to the high expression of certain amino acids, predict the function of genes and analyze the phylogeny and evolution of species [23,24]. Relative synonymous codon usage (RSCU) reflects the ratio of the frequency of usage of a codon to the expected frequency and is a measure of nonuniform synonymous codon usage in coding sequences [22,25]. The RSCU values of 1.6 represent over-represented codons, whereas codons that are less than 0.6 are under-represented, the RSCU values that fall between 1.6 and 0.6 indicate codons with no bias or are not randomly used [26]. A total of 24 codons were biased codons (Figure 3). Among these 24 biased codons, 9 codons were found in non-turtle species, with 5 codons having RSCU values greater than 1.6 and 4 codons having RSCU values less than 0.6. Only one codon was biased used in the turtle species *R*. *swinhoei*. Another 14 biased codons shared among turtles and other detected species, with 4 codons having RSCU values greater than 1.6 and 10 codons having RSCU values less than 0.6 (Figure 3).

### 3.4. Duplicate Genes in Turtles

**Figure 4:**
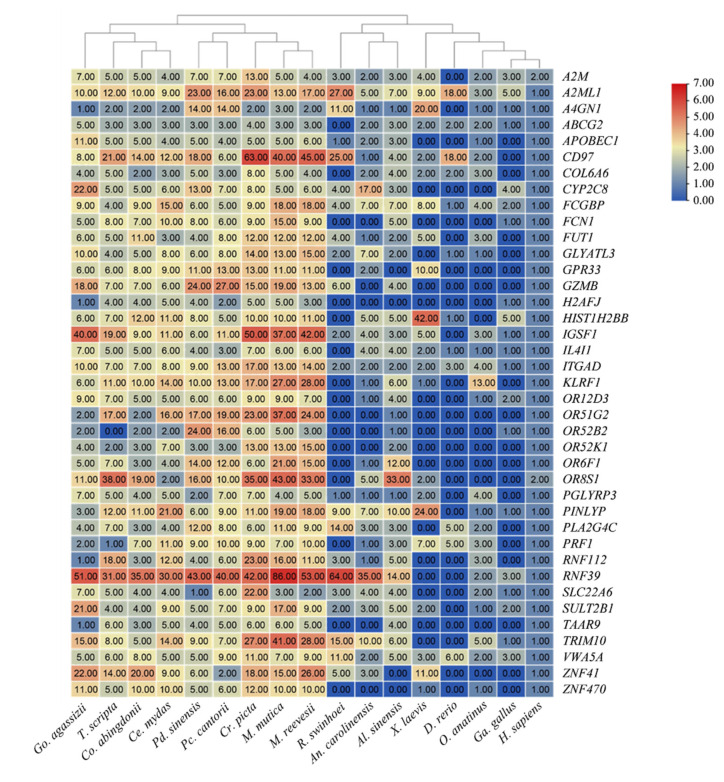
Duplicate genes among 17 phylogenomically analyzed 17 vertebrates. Blue to red indicates the number of gene copies from low to high.

Duplicate genes have long been regarded as important drivers of novel functions and adaptive evolution [27,28,29]. We compared the duplicated genes among 17 vertebrate species, including *Cr. picta*, *M. mutica*, *M. reevesii*, *Go. agassizii*, *Pc. cantorii*, *T. scripta*, *Chelonoidis abingdonii*, *Ce. mydas*, *R. swinhoei*, *An. carolinensis*, *Al. sinensis*, *X. laevis*, *D. rerio*, *O. anatinus*, *Pd. sinensis*, *Ga. gallus* and *H. sapiens.* There are 39 genes with multiple copy numbers in *Pc. cantorii*, and most of these genes also have a higher copy number in most identified turtles such as *Cr. picta*, *M. mutica*, *M. reevesii*, *Go. agassizii*, *T. scripta*, *Co. abingdonii* and *Ce. mydas* (Figure 4). Subsequently, a tumor-suppressor gene database (TSGs) was used to annotate the selected candidate genes and then obtain clearly defined tumor-suppressor genes, including *von Willebrand Factor A domain containing 5A* (*VWA5A*) and *ATP binding cassette subfamily G member 2* (*ABCG2*) (Figure 4). Moreover, the Aging Gene Database (GenAge) was used to annotate the screened candidate genes and acquire age-related genes. We found that seven copies of *alpha-2-macroglobulin* (*A2M*) were revealed in *Pc. cantorii*. *A2M* is known to dramatically decrease with age in humans [30], and the exposure of tumor cells to activated *A2M* inhibits many malignancy-associated properties of tumor cells in vitro by inhibiting members of the WNT/β-catenin pathway [31]. Notably, among these duplicated genes, we found a body-size-related gene, *immunoglobulin superfamily member 1* (*IGSF1*), which is associated with central hypothyroidism [32], DNA damage and telomere-stress-induced aging [33].

## 4. Discussion

The Asian giant soft-shelled turtle, one of the largest aquatic turtles, was once widely distributed in Southeast Asia. Their existence is currently threatened because of anthropogenic activities, such as overhunting and destruction of habitats. Therefore, it is necessary to construct strategies for conserving and managing the current individuals, and the establishment of a high-quality genome is a prerequisite for the conservation of *Pc. cantorii*. In our previous study, we combined third-generation PacBio sequencing with Hi-C scaffolding technologies to develop a high-quality chromosome-level genome and annotations for this threatened species. We obtained 262.77 Gb of clean data, which represented approximately 121.6 × coverage of the *Pc. cantorii* genome. The assembled genome comprised 2.16 Gb with a contig N50 of 41.44 Mb and scaffold N50 of 120.17 Mb. Moreover, the Hi-C scaffolding of the genome ordered onto 33 chromosomes (Chr), accounting for 99.98% of the total assembly, which was consistent with the karyotype analyses of *Pc. cantorii* (scientific data under review). The chromosomal-level genome provides important resources for extensive studies on the genetic basis and germplasm conservation of the Asian giant soft-shelled turtle.

Comparative genomics analysis of 17 vertebrate species, including *Pc. cantorii*, *Pd. sinensis*, *R. swinhoei*, *X. laevis*, *Al. sinensis*, *Ga. gallus*, *O. anatinus*, *An. carolinensis*, *D. rerio* and *H. sapiens*, revealed that *Pc. cantorii* was most closely related to *Pd. sinensis* and R. swinhoei, and was then clustered with T. scripta, Ce. mydas, Cr. picta, M. mutica, *M. reevesii*, *P. megacephalum* and *Go. agassizii*, which demonstrated a common ancestor shared between hard- and soft-shelled turtles (Figure 1). Additionally, the phylogenetic relationships constructed on a set of turtle orthologs previously indicated that turtles are likely to be a sister group of archosaurs (alligator plus birds) [34,35,36]. A phylogenetic reconstruction in our genome-scale analyses also placed turtles as well nested within diapsid amniotes (Figure 1). Analysis of time-constrained molecular clocks based on the fossil record shows that turtles separated from the ancestor of archosaurians approximately 256.6 (263.6–251.9) Mya (Upper Permian to Triassic). The overlapping of the Permian extinction event may have caused the emergence of the turtle group [37]. The hard- and soft-shelled turtles split 164.4 (181.7–147.3) Mya (Figure 1), and the earliest known turtle fossil is the Late Triassic Proganochelys fossil dating back 200 million years [38]. The Jurassic period was an early stage in the evolution of tortoises, and it is possible that at this time, soft-shelled turtles accustomed to freshwater habitats began to diverge from hard-shelled groups, such as sea turtles or land turtles.

Moreover, we compared the *Pc. cantorii* genome with the *R. swinhoei*, *Ce. mydas*, *Cr. picta*, *M. mutica*, *M. reevesii*, *T. scripta* and *Ga. gallus* genomes to further examine the chromosome evolution events after speciation. We found that twenty-six chromosomes of *Pc. cantorii* were aligned unambiguously to single chromosomes of *R. swinhoei* (Figure 2A). Previous studies indicate that *Dmrt1* and *Amh*, which play important roles in sex differentiation and development, are located on chr2 of *R. swinhoei*, suggesting that chr2 may be (part of) the potential sex chromosomes *R. swinhoei* [36]. Here, chr2 in *R. swinhoei* was corresponded to chr17 in *Pc. cantorii* (Figure 2A). Chr5 of *Pc. cantorii* mapped to Z chromosome of *Ga. gallus* (Figure 2G). These results indicated that *Pc. cantorii* may also have a ZZ/ZW sex determination system.

Codon usage bias influences the function of the protein and its translation efficiency, which is an important event for molecular evolutionary phenomena, such as mutation, selection, and random genetic drift [39,40,41]. Generally, codon usage patterns vary among species, genes of the same species may adopt similar codon selection strategies [42]. Moreover, species with closer phylogenetic relationships or similar living environment may have similar codon usage patterns. Here, compared with other species, including *O. anatinus*, *X. laevis*, *An. carolinensis*, *Al. sinensis*, *D. rerio*, *H. sapiens* and *Ga. gallus*, only one codon was biased used in the turtle species *R*. *swinhoei*. In total, 10 out of 14 based codons are shared in turtles and other detected species having RSCU values less than 0.6 (Figure 3). The codon preference is mainly formed by species for high expression of certain amino acids, perhaps because there is no need to have an extreme preference for certain amino acids or at least not in turtle species as extreme as other species identified in this study.

Moreover, many turtle species live 100 years or more and are an ideal model to investigate the mechanism of longevity [43]. Duplicated genes are considered raw materials for evolutionary innovations [44]. In the present study, there are 39 genes with multiple copy numbers in *Pc. cantorii* (Figure 4). Notably, among these duplicate genes, we characterized several candidates associated with tumor suppression, growth and age based on database annotation, which might play a significant role in exceptional longevity. *VWA5A*, also known as *BCSC-1* or *LOH11CR2A*, may be useful as a biomarker for the treatment of breast cancer and has been revealed to suppress human-breast-cancer cell migration and invasion, potentially altering the expression of MMP7, MMP9 and OPN, and the activity of the NF-κB pathway [45]. *ABCG2*, also known as breast-cancer resistance protein (BCRP), is a multidrug-resistant protein that is a member of the ATP-binding cassette family of drug transporters [46]. More copy numbers of *VWA5A and ABCG2* identified in turtles may provide mainly function in immunity and metabolism, which will be associated with turtle longevity. Moreover, we also examined the age-related gene *A2M* and body-size-related gene *IGSF1*. Specifically, *A2M* encodes a proteinase inhibitor that binds with Aβ peptides tightly and prevents the formation of Aβ plaques in the brains of Alzheimer’s disease patients [47]. The multiple copies of *A2M* in turtles are likely to be important for the longevity of these species. *IGSF1* has been shown to be associated with the regulation of thyroid hormone, and the expression levels of *IGSF1* are correlated with thyroid cancer cell growth, metastasis and apoptosis [48,49]. Moreover, *IGSF1* is also a strong candidate for body size [50] and plays significant roles in modulating weight variation and contributing to the muscled phenotype [51], while there is a significant interaction between body size and aging in eukaryotes, especially vertebrates [52,53]. Therefore, the identification of more copy numbers of these genes will provide important insights into future studies to explore the longevity mechanism of *Pc. cantorii*, and their roles remain to be further explored.

## 5. Conclusions

Based on a combination of Illumina short-read, PacBio long-read and Hi-C scaffolding technologies, we previously obtained a high-quality chromosome-level genome of *Pc. cantorii*. In this study, we first explored the evolutionary state of *Pc. cantorii* by the analysis of phylogenetic relationship among 17 vertebrates. We found that the overlapping of the Permian extinction event may have raised the emergence of the turtle group, and soft-shelled turtles accustomed to freshwater habitats possibly split from hard-shelled turtles, such as sea turtles or land turtles, during the Jurassic period. Moreover, we also identified several expanded candidate genes, such as *VWA5A*, *ABCG2*, *A2M* and *IGSF1,* which are associated with tumor suppression, growth and age. Therefore, more biological characteristics of *Pc. cantorii* will be investigated based on this new chromosome-level assembly.

## Data Availability

The sequencing data have been deposited in the NCBI Sequence Read Archive database under the BioSample accession numbers. The accession number of coding sequences, Illumina, PacBio, gene, exon, Hi-C and full-length transcriptome sequences were SRR22715189, SRR22681424-SRR22681426, SRR22296394, SRR22715197 and SRR22674657, SRR23047442 and SRR23047393, respectively.
